# Aging exacerbates development of cerebral microbleeds in a mouse model

**DOI:** 10.1186/s12974-018-1092-x

**Published:** 2018-03-06

**Authors:** Rachita K. Sumbria, Mher Mahoney Grigoryan, Vitaly Vasilevko, Annlia Paganini-Hill, Kelley Kilday, Ronald Kim, David H. Cribbs, Mark J. Fisher

**Affiliations:** 1 Department of Biopharmaceutical Sciences, School of Pharmacy, Keck Graduate Institute, Claremont, CA USA; 2 Department of Neurology, University of California, Irvine, CA USA; 3 Institute for Memory Impairments and Neurological Disorders, University of California, Irvine, CA USA; 4 Department of Pathology and Laboratory Medicine, University of California, Irvine, CA USA; 5 Department of Anatomy and Neurobiology, University of California, Irvine, CA USA; 6 UC Irvine Medical Center, 101 The City Drive South, Shanbrom Hall, Room 121, Orange, CA 92868 USA

**Keywords:** Animal models, Cerebral microhemorrhage, Cerebral microbleeds, Inflammation, Hemosiderin, Aging

## Abstract

**Background:**

Cerebral microhemorrhages (CMH) are commonly found in the aging brain. CMH are also the neuropathological substrate of cerebral microbleeds (CMB), demonstrated on brain MRI. Recent studies demonstrate the importance of systemic inflammation in CMH development, but the relationships among inflammation, aging, and CMH development are not well-defined. In the current study, we hypothesized that the pathogenesis of inflammation-induced CMH in mice differs by age.

**Methods:**

We studied young (3 months, *n* = 20) and old (18 months, *n* = 25) C57BL/6 mice injected with low-dose lipopolysaccharide (LPS; 1 mg/kg, i.p.) or saline at 0, 6, and 24 h. Seven days after the first LPS/saline injection, brains were harvested, sectioned, and stained with hematoxylin and eosin (H&E) and Prussian blue (PB) to estimate acute/fresh and sub-acute CMH development, respectively. The relationships between microglial/macrophage activation (ionized calcium-binding adapter molecule-1), astrocyte activation (glial fibrillary acidic protein), blood-brain barrier (BBB) disruption (brain immunoglobulin G), aging, and CMH development were examined using immunohistochemistry.

**Results:**

Aging alone did not increase spontaneous H&E-positive CMH development but significantly increased the number, size, and total area of LPS-induced H&E-positive CMH in mice. LPS- and saline-treated aged mice had significantly larger PB-positive CMH compared with young mice, but the total area of PB-positive CMH was increased only in LPS-treated aged mice. Aged mice had significantly increased microglial/macrophage activation, which correlated with H&E- and PB-positive CMH development. Aged mice treated with LPS had significantly increased astrocyte activation and BBB disruption compared with young LPS-treated mice.

**Conclusions:**

Aging makes the brain more susceptible to inflammation-induced CMH in mice, and this increase in CMH with aging is associated with microglial/macrophage activation.

## Background

Age is the most significant independent risk factor for cerebral microbleeds (CMB), which are identified by brain MRI and have as their pathologic substrate cerebral microhemorrhages (CMH) [1, 2]. In addition to aging, CMB are associated with hypertension, cerebral amyloid angiopathy (CAA), Alzheimer’s disease, cerebral autosomal-dominant arteriopathy with subcortical infarcts and leukoencephalopathy (CADASIL), and chronic kidney disease, among other diseases [3–7]. Despite their high prevalence and clinical importance, the mechanisms underlying age-related CMB increase are not well-understood. One mechanism suggested by clinical findings is the activation of inflammatory cascades followed by blood-brain barrier (BBB) damage resulting in CMH development [8–11]. In fact, a pro-inflammatory systemic state is a common factor observed in normal aging and diseases with a high prevalence of CMB [12–15].

Recently, we showed that systemic inflammation induced by lipopolysaccharide (LPS) administration results in CMH development in mice and that CMH development was associated with levels of systemic and neuroinflammatory markers [2, 16]. However, the relationships among aging, inflammation, and CMH development are not well-defined [3]. The current study aimed to determine if aging exacerbates inflammation-induced CMH, and we hypothesized that the pathogenesis of inflammation-induced CMH in mice differs by age. We utilized the inflammation-induced CMH mouse model which results in MRI-demonstrable CMB [2]. LPS was used as the inflammatory stimulus to induce CMH development in young (3-month old) and aged (18-month old) mice. Age-related changes in both hematoxylin and eosin (H&E)-positive (acute) and Prussian blue-positive (sub-acute) CMH were examined in relationship to markers of neuroinflammation and BBB damage.

## Methods

### Mouse treatment

All animal procedures were approved by the University of California, Irvine, Institutional Animal Care and Use Committee, and followed the ARRIVE Guidelines for animal experiments reporting. Young (3-month old) and aged (18-month old) C57BL/6 male and female mice (National Institute of Aging, Bethesda, MD) were randomly assigned to two treatment groups: one treated with a 1 mg/kg dose of LPS derived from gram-negative bacterium *Salmonella typhimurium* (Sigma-Aldrich, St. Louis, MO) and the other treated with saline intraperitoneally (i.p.) at three times at 0, 6, and 24 h. Seven days after the first LPS/saline injection, mice were anesthetized with a lethal dose of Nembutal (150 mg/kg, i.p.), cardiac perfusions were performed using ice-cold PBS, and brains were processed for CMH detection as described in our previous work [16]. The sample size for each group was as follows: young-saline = 10, aged-saline = 10, young-LPS = 10, and aged-LPS = 15. The average weight of the mice was 28.1 ± 0.9 g at the beginning of the study and 27.1 ± 0.8 at the end of the study.

### Microhemorrhage detection

Right hemispheres were fixed in 4% paraformaldehyde (Boston BioProducts, Ashland, MA) at 4 °C, examined for surface CMH, and sectioned into 40-μm coronal sections using a vibratome (Technical Products International, Inc., St. Louis, MO). Every fourth, fifth, sixth, and seventh section was collected. Every sixth section was stained with H&E by Research Services Core offered by the Department of Pathology and Laboratory Medicine at UCI Medical Center to detect fresh (acute) CMH. Every seventh section was stained with Prussian blue (PB) to detect hemosiderin (a marker of sub-acute CMH) [16]. Briefly, sections were stained using 5% potassium hexacyanoferrate trihydrate (Sigma, St. Louis, MO) and 5% hydrochloric acid (Sigma, St. Louis, MO) for 30 min, rinsed in water and counterstained with Nuclear Fast Red (Sigma, St. Louis, MO), dehydrated, and cover slipped. Remaining sections were used for immunohistochemistry. CMH were counted at a × 20 magnification by a blinded observer, and digitized images were used to determine CMH size (μm^2^) and positive area (expressed as a percent of total area analyzed), by an observer blinded to the experiment using RGB CMH analyzer program and NIH Image J software 1.62, respectively [16].

### IgG, Iba-1, and GFAP immunohistochemistry

Parenchymal IgG (BBB damage marker), Iba-1 (microglial/macrophage marker), and GFAP (astrocyte marker) immunohistochemistry were performed [16]. Briefly, 40-μm sections were incubated in 0.5% hydrogen peroxide in 0.1 M PBS (pH 7.4) containing 0.3% Triton-X100 (phosphate-buffered saline with triton-X100 (PBST)) for 30 min at room temperature (RT), washed with PBST, and blocked with PBST containing 2% bovine serum albumin for 30 min at RT. Sections were then incubated overnight at 4 °C with a rabbit anti-mouse IgG antibody (1:200 dilution; Jackson ImmunoResearch, West Grove, PA), rabbit antibody against Iba-1 (1:200 dilution, Wako Chemicals USA, Richmond, VA), or rabbit antibody against GFAP (1:2000 dilution, Abcam, Cambridge, MA). After washing with PBST, sections were incubated at RT for 1 h with biotinylated anti-rabbit IgG (1:500 dilution; Jackson ImmunoResearch, West Grove, PA), followed by 1 h incubation at RT with ABC complex, and developed with 3,3′-diaminobenzidine (DAB) (Vector Laboratories, Burlingame, CA) as per manufacturer’s instructions. Sixteen images per brain section were acquired at × 20 magnification, and the total positive immunoreactive area (expressed as percent of total analyzed area) was quantified using NIH Image J software 1.62 by an observer blinded to the experimental groups [16].

### Statistical analysis

Based on our previous work showing a mean number of CMH per brain section of 1.3 ± 0.8 in the LPS group and 0.03 ± 0.05 in the saline group, we estimated that at least 10 mice per group were needed to detect a significant difference between LPS and control mice at 80% power. One-way ANOVA with Bonferroni’s post hoc test for normal data and Kruskal-Wallis with Dunn’s multiple comparison test for non-normal data were used to compare more than two groups. Two-way ANOVA with Tukey’s post hoc test was used for sex comparisons. Pearson *r* correlation was used for correlation analysis. One-sample *t* test was used to compare group means with a hypothesized mean = 0 when the values in the control group were zero (e.g., surface microhemorrhages). A two-tailed *p* value of < 0.05 was considered statistically significant.

## Results

### Survival

All the saline-treated young and aged mice and LPS-treated young mice survived the duration of the study (*n* = 10 per group). Four aged mice treated with LPS died prematurely reducing the number included in the analysis to 15.

### Cerebral microhemorrhages

**Fig. 1:**
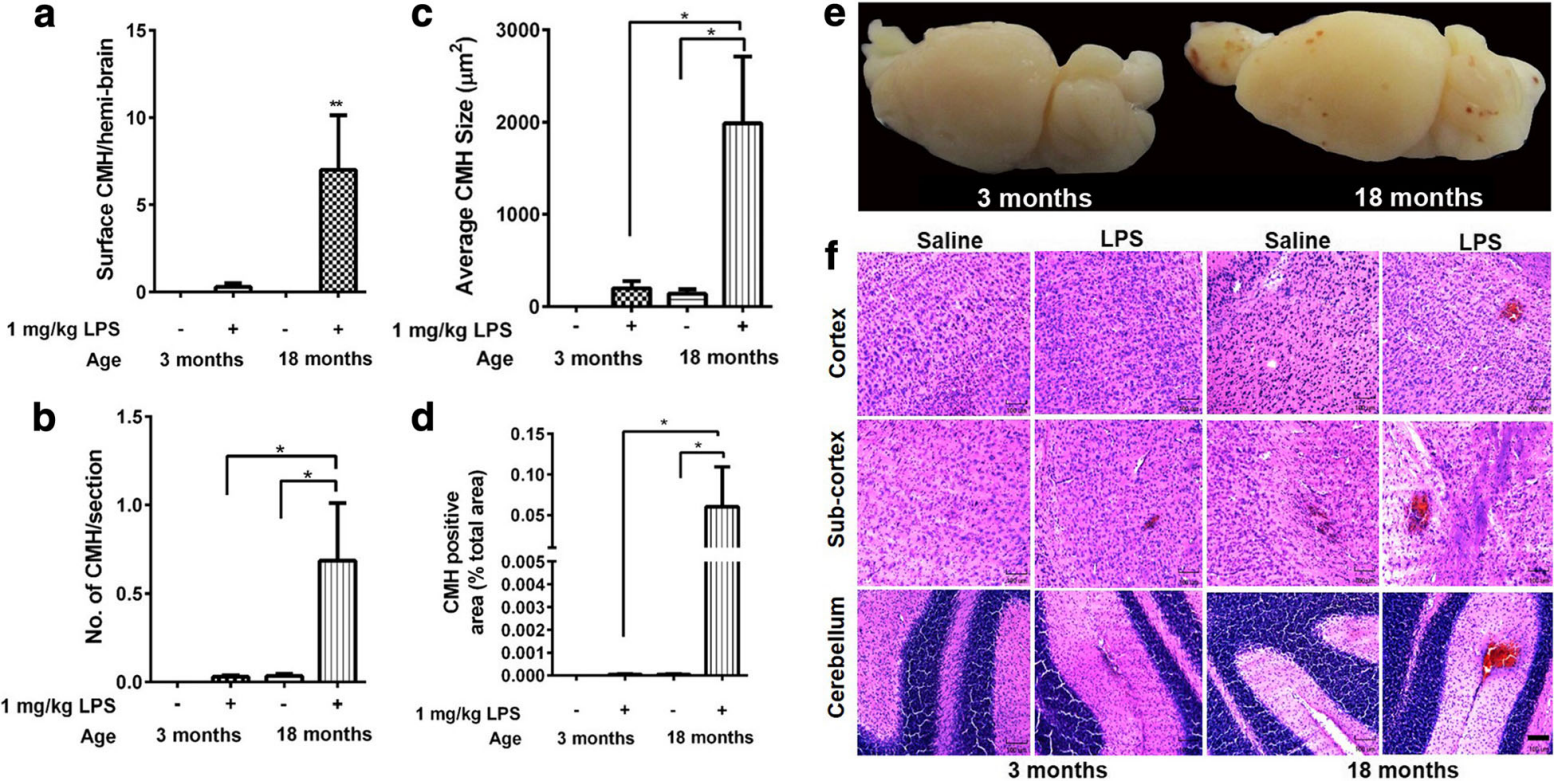
Surface and acute H&E-positive cerebral microhemorrhages (CMH). No development of surface CMH in young and aged saline-treated mice (*n* = 10 per group). Significant development of surface CMH in aged LPS-treated mice (*n* = 15) compared with young LPS-treated (*n* = 10) and aged saline-treated mice (*n* = 10) (**a**). No development of acute (H&E-positive) parenchymal CMH in saline-treated young mice. Significantly greater number, size, and total area of acute parenchymal CMH in LPS-treated aged mice compared with LPS-treated young mice and saline-treated aged mice (**b**–**d**). Brain images showing development of surface CMH in LPS-treated aged mice compared with LPS-treated young mice (**e**). Representative images showing acute CMH in different brain regions (**f**). Data are presented as mean ± SEM. One-sample *t* test for surface CMH, one-way ANOVA with Bonferroni’s post-test, or Kruskal-Wallis test with Dunn’s post-test. *p < 0.05 and **p < 0.01. Scale bar = 100 μm

Aging exacerbated the development of LPS-induced surface CMH, and the number of surface CMH in LPS-treated aged mice increased 23-fold compared with LPS-treated young mice (Fig. 1a, e). No surface CMH were observed in saline-treated mice, young or old.

In young mice, no spontaneous (in the absence of LPS) H&E-positive acute CMH were observed in saline-treated mice and a 1 mg/kg triple dose of LPS did not result in significant development of H&E-positive acute CMH (Fig. 1b–d, f). Aging did not cause a significant increase in spontaneous acute CMH development in saline-treated mice; however, similar to the effect on surface CMH development, aging significantly increased the development of H&E-positive acute parenchymal CMH in LPS-treated mice (Fig. 1b–d, f). All three parameters (number, size, and total area) of H&E-positive acute parenchymal CMH were significantly increased with age in LPS-treated mice (Fig. 1b–d). H&E-positive lesions were significantly higher in the cerebellum compared with the cortex and the sub-cortex (data not shown).

**Fig. 2:**
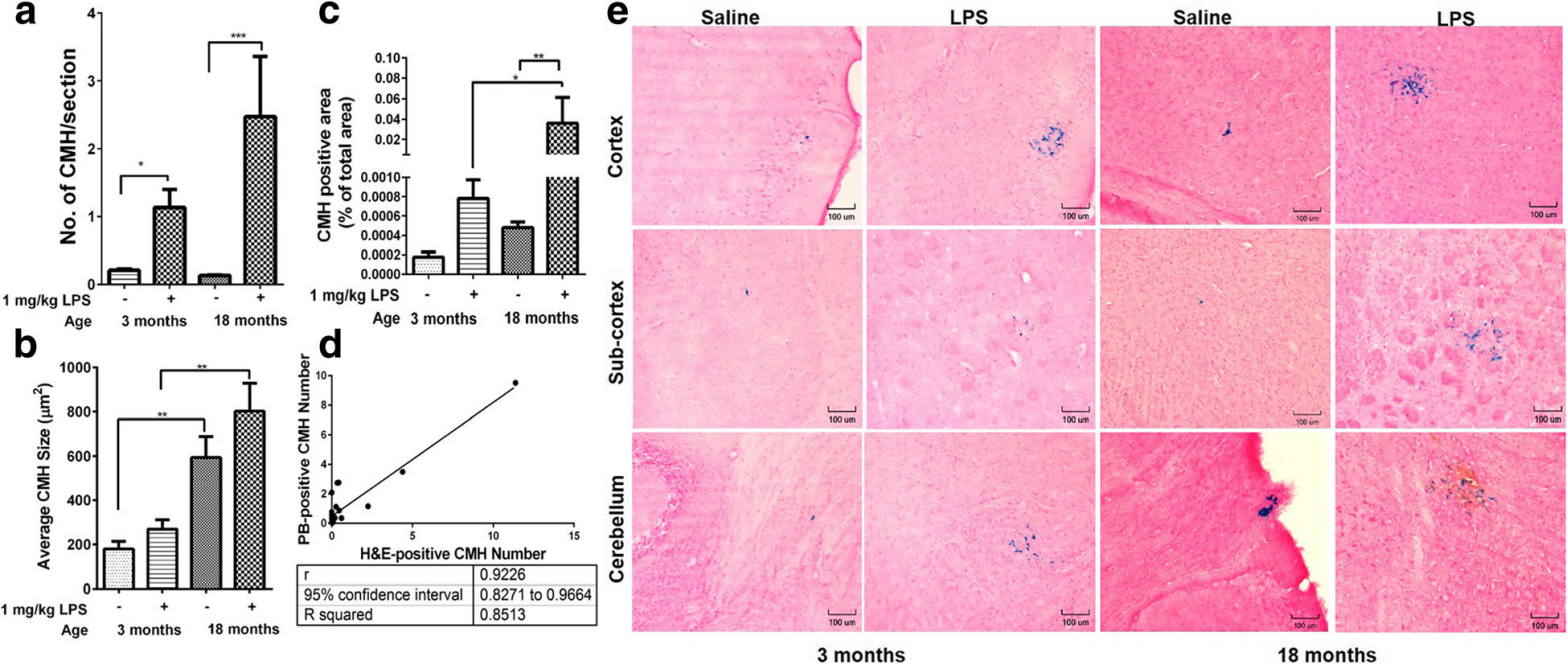
Sub-acute Prussian Blue (PB)-positive cerebral microhemorrhages (CMH). Significantly higher number of PB-positive lesions in LPS-treated young (*n* = 10) and LPS-treated aged mice (*n* = 15) compared with their respective saline controls (*n* = 10 per group) (**a**). Significant increase in PB-positive lesion size with aging (**b**). Significant increase in PB-positive total lesion area in LPS-treated aged mice compared with LPS-treated young mice and saline-treated aged mice (**c**). PB-positive and H&E-positive lesion number are significantly correlated in aged mice (**d**). Representative images showing PB-positive stains in different brain regions (**e**). Scale bar = 100 μm. Data are presented as mean ± SEM. One-way ANOVA with Bonferroni’s post-test or Kruskal-Wallis test with Dunn’s post-test; *p < 0.05, **p < 0.01, and ***p < 0.001

The number of PB-positive sub-acute parenchymal CMH was significantly higher in the LPS-treated young and LPS-treated aged mice compared with their respective saline controls (Fig. 2a–c). The number of PB-positive CMH lesions increased twofold in LPS-treated aged mice compared with LPS-treated young mice, although this increase did not reach statistical significance. Aging independently (with and without LPS) caused a significant increase in PB-positive lesion size; average size increased approximately threefold in both saline- and LPS-treated aged mice compared with their respective young controls (Fig. 2b). Total PB-positive lesion area, which is a function of both lesion number and size, was significantly increased with age in LPS-treated mice (Fig. 2c). The number of H&E-positive acute and PB-positive sub-acute lesions were significantly correlated (Pearson *r* = 0.92, *p* < 0.0001) in aged mice (Fig. 2d) but not in young mice (data not shown).

### Relationship between CMH development, neuroinflammation, BBB damage, and aging

**Fig. 3:**
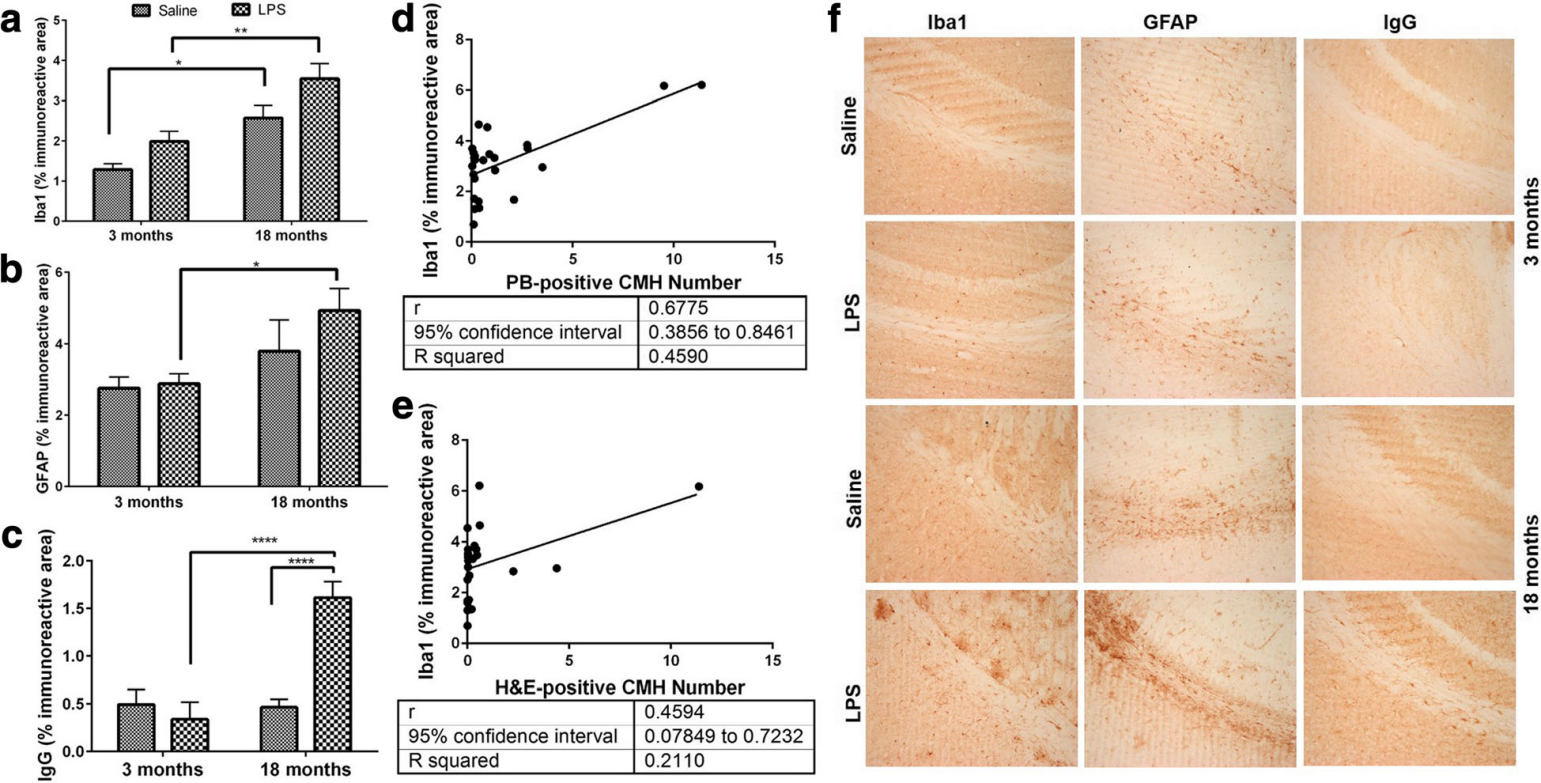
Neuroinflammation, BBB disruption, and CMH development. Significantly higher Iba-1-positive immunoreactivity with aging (**a**), and GFAP- (**b**) and IgG- (**c**) positive immunoreactivity in LPS-treated aged mice compared with LPS-treated young mice. PB- and H&E-positive CMH numbers are significantly correlated with Iba-1 positive immunoreactivity in aged mice (**d**, **e**). Representative images of Iba-1, GFAP, and IgG immunohistochemistry (**f**). Data are presented as mean ± SEM. One-way ANOVA with Bonferroni’s post-test or Kruskal-Wallis test with Dunn’s post-test and Pearson correlation; *p < 0.05, **p < 0.01, and ****p < 0.0001

Aging was associated with a significant increase in brain Iba-1-positive immunoreactivity, independent of LPS treatment (Fig. 3a). Immunoreactive area of the astrocyte activation marker (GFAP) and BBB damage marker (brain IgG) on the other hand were significantly increased with age only in LPS-treated mice (Fig. 3b, c). Overall, both the H&E-positive and PB-positive lesion numbers were significantly correlated with Iba-1-positive immunoreactivity (Fig. 3d, e).

### Sex and CMH development

In an exploratory analysis of the relationship between sex and CMH development, the number (*p* < 0.01), size (*p* < 0.0001), and total area (*p* < 0.01) of H&E-positive acute parenchymal CMH were increased with age in LPS-treated male mice compared with LPS-treated female aged mice. No sex differences were observed in PB-positive CMH and in markers of neuroinflammation and BBB injury among young or aged mice (data not shown).

## Discussion

In the current study, we investigated the effect of aging on inflammation-induced CMH development in a mouse model. We demonstrated negligible spontaneous acute CMH development in saline-treated young and aged mice. Exacerbation of acute CMH development was observed in LPS-treated aged mice and not in LPS-treated young mice. Since H&E staining enables the detection of fresh or acute microhemorrhages in the brain [2], the presence of fresh microhemorrhages in aged LPS-treated mice suggests that inflammation-induced CMH development is an ongoing process that lasts days after the last LPS injection in aged mice but not young mice (last LPS injection at day 2 and sacrifice at day 7 in the current study). PB on the other hand detects hemosiderin that remains at the bleeding site for a prolonged period of time, thus enabling the detection of cumulative CMH load at the time of sacrifice.

LPS treatment resulted in significant development of PB-positive sub-acute CMH in both young and aged mice. Consistent with other findings and our own work, we found low numbers of spontaneous PB-positive CMH in young and aged mice [2, 17]. Total PB-positive CMH load was significantly higher in LPS-treated aged mice and not in LPS-treated young mice. Spontaneous PB-positive CMH were significantly larger in aged mice, but this effect was independent of LPS treatment. The number of PB-positive lesions was highly associated with the number of H&E-positive lesions (*r*^2^ = 0.85) further suggesting that hemosiderin-positive sub-acute microhemorrhages detected by PB may help predict the susceptibility of the aging brain to develop acute or fresh microhemorrhages. Taken together, these results show that aging and inflammation together make the brain more vulnerable to CMH development and further corroborate previous studies that support the role of inflammation in the pathogenesis of CMH [8, 9, 18].

Though the exact molecular mechanisms underlying inflammation-induced CMH development are not completely understood, loss of microvascular integrity associated with inflammatory changes is associated with CMH development [19]. In the current study, we observed an increase in BBB disruption in LPS-treated aged mice consistent with our previous work [16]. Systemic administration of LPS is known to result in BBB injury and inflammation by altering tight junction protein expression, enlarging intercellular clefts, and increasing cytokine and chemokine release [20, 21]. These effects of LPS are mediated by its binding to toll-like receptor 4 (TLR4), which activates signaling pathways implicated in BBB disruption [22]. The contribution of TLR4 in intracerebral hemorrhage (ICH)-induced brain injury and inflammation has been documented [23], and a recent study showed that endothelial TLR4 activation by its canonical ligand LPS accelerates cerebral cavernous malformations (CCM) [24]. Given the role of TLR4 activation in ICH and CCM development and increased expression of TLR4 with the LPS dose used in the current study [25], TLR4 activation via LPS may mediate at least some of the effects observed in the current study. Overall, increased LPS-mediated BBB disruption in the current study further supports the role of the inflammation-induced loss of BBB integrity in CMH development [11]; the underlying role of TLR4 in CMH development needs further investigation. Loss of BBB integrity detected at the time of sacrifice in the aged mice in the current study may further explain the presence of acute CMH several days after the last LPS injection in aged mice and not LPS-treated young mice.

Studies show that hemosiderin deposits in the brain are often surrounded by macrophages which can further initiate an inflammatory response [26]. Consistent with this and our previous work, we found a significant increase in two different markers of neuroinflammation, Iba-1 (microglial/macrophage activation marker) and GFAP (astrocyte activation marker), in aged mice [2]. Iba-1 immunoreactivity was elevated with aging in the current study and was independent of LPS treatment. Further, we found that microglia/macrophage activation was significantly associated with CMH development in aged mice. We also found an increase in astrocyte activation in the aged mice treated with LPS, further indicating the increased susceptibility of the aging brain to neuroinflammation.

The role of microglia/macrophages in the pathogenesis of CMH is not well-defined. Apart from exhibiting a cytotoxic phenotype (M1 phenotype), microglia/macrophages can also mediate repair mechanisms in the brain depending on the brain microenvironment (M2 phenotype) [27, 28]. M2 macrophages are associated with hematoma resolution after intracerebral hemorrhage in a mouse model [29]. Whether the macrophage/microglial response to CMH observed in the current study is protective (clearing the CMH) or detrimental (triggering local inflammation) needs further investigation.

Sex differences in CMB development have been reported in clinical settings and CMB are more prevalent in males [30]. An exploratory analysis of our data showed that LPS-treated aged male mice were more susceptible to acute (H&E-positive) CMH development compared to LPS-treated aged female mice. We observed no sex differences in subacute (PB-positive) CMH development in aged mice. These sex differences in LPS-induced CMH development are consistent with prior observations on sex differences in response to LPS [31]. We speculate that inflammation-induced CMH development is an ongoing process in aged male mice at the time of sacrifice (7 days), compared with aged female mice. Future experimental studies are needed to fully define this process.

## Conclusions

The current study highlights the relationships among aging, inflammation, and CMH development. Aging increases the susceptibility of the brain to inflammation-induced CMH development in mice, and this increase in CMH development is significantly associated with microglial/macrophage activation. The exact role of microglial activation in CMH pathogenesis needs further investigation.

## Abbreviations

BBB: Blood-brain barrier
CAA: Cerebral amyloid angiopathy
CADASIL: Cerebral autosomal-dominant arteriopathy with subcortical infarcts and leukoencephalopathy
CCM: Cerebral cavernous malformations
CMB: Cerebral microbleeds
CMH: Cerebral microhemorrhages
DAB: 3,3′-Diaminobenzidine
GFAP: Glial fibrillary acidic protein
H&E: Hematoxylin and eosin
Iba-1: Ionized calcium-binding adapter molecule-1
ICH: Intracerebral hemorrhage
IgG: Immunoglobulin G
LPS: Lipopolysaccharide
PB: Prussian blue
